# Applying co-design health literacy development in Australian prisons: protocol for system-wide application of the Optimising Health Literacy and Access (Ophelia) process

**DOI:** 10.1136/bmjopen-2024-092128

**Published:** 2025-04-07

**Authors:** Scott W Gill, Julia Bowman, Christina Cheng, Caron Shaw, Stephen Hampton, Wendy Hoey, Richard H Osborne

**Affiliations:** 1 Centre for Global Health and Equity, School of Health Sciences, Swinburne University of Technology, Hawthorn, Victoria, Australia; 2 Justice Health and Forensic Mental Health Network, Matraville, New South Wales, Australia; 3 Clinical Excellence Commission, Sydney, New South Wales, Australia; 4 Department of Public Health, University of Copenhagen, Kobenhavn, Region Hovedstaden, Denmark; 5 NOVA University of Lisbon, Lisboa, Portugal

**Keywords:** Prisons, Health Literacy, Organisational development, Health Services, Health Equity, PUBLIC HEALTH

## Abstract

**Abstract:**

**Introduction:**

Prisons present both unique opportunities and challenges for delivering healthcare to individuals who often experience significant vulnerabilities and often have poor health outcomes. Actions and solutions informed by the health literacy strengths and challenges (ie, health literacy-informed interventions) of people in prison offer an opportunity to build fit-for-purpose and effective interventions in this unique context. This study aims to adapt and apply the three-phase Optimising Health Literacy and Access (Ophelia) process in a state-wide prison context to generate codesigned improvements in information, resources and services for people in prison.

**Methods and analysis:**

Health Literacy Questionnaire data from 471 people in prison will be analysed using descriptive and cluster analyses (Ophelia Phase 1). Clusters, with qualitative interview data, will then inform vignette development for use in ideas generation workshops and yarning circles with stakeholders to develop health literacy-informed interventions. Selection, prioritisation and testing of identified interventions will be undertaken (Phase 2), followed by implementation and evaluation (Phase 3). This project will advance intervention development in the prison context, enabling the voice of people in prison and service providers to be heard through codesign. The protocol will inform the development and implementation of interventions to systematically improve the delivery of information, services and resources for people in prison, which may be relevant to prison healthcare authorities globally.

**Ethics and dissemination:**

Ethical approval to undertake Phase 1 of the Ophelia process has been granted from the following Human Research Ethics Committees: Swinburne University of Technology (Ref: 20236977–15461), Justice Health NSW (Ref: 2022/ETH01433), Aboriginal Health and Medical Research Council (Ref: 2007/22) and the Corrective Services Ethics Committee (Ref: D2022/1452326). Dissemination of the study findings will be the Justice Health NSW codesign process and ownership of the project through authentic engagement with people with lived experience and health and corrective staff. It will also be disseminated through publication in a PhD thesis, peer-reviewed research papers and conference presentations.

**STRENGTHS AND LIMITATIONS OF THIS STUDY**

- The Health Literacy in Prison Project will use the Optimising Health Literacy and Access (Ophelia) process, a well-documented and proven approach to equitable health services improvement.
- The use of bottom-up and top-down stakeholder engagement processes will enhance the development of fit-for-purpose and effective health literacy-informed solutions in a context where people experience substantial vulnerabilities.
- A strength of this study is the authentic codesign process that will engage people in prison, health and corrective staff in harnessing local wisdom to collaboratively develop actions and solutions to improve access to health information and services.
- A weakness of this protocol is limited engagement with non-English speakers, those who may not access healthcare and those in rural or remote prisons, which may result in the needs assessment not fully capturing the experiences of all people in New South Wales prisons.
- A potential limitation is the absence of people who feel unsafe in group settings or cannot provide informed consent to participate in ideas generation workshops and later study phases.

## Background

 Prisons are not conducive environments for health.^1^,^3^ Prisons are institutions of the criminal justice system designed to restrict an individual’s liberty for a period as determined by a judicial authority (ie, the courts).^4^ The role of these institutions is to protect society and rehabilitate an individual for their return to the community.^5 6^ Over the past 50 years, political agendas, such as the ‘war on drugs’^7^ and reforms such as mandatory sentence lengths, have put considerable strain on criminal justice systems globally.^8^ Additionally, safety and security take precedence in prisons, creating structural barriers to engaging with or providing healthcare services.^9 10^

### People in prison

People in prison experience a wide range of vulnerabilities and poorer health outcomes compared with the general populations.^11 12^ Globally, it is estimated that 11 million people are incarcerated.^13^ At any given time, over 42 000 people are in Australian prisons.^14^ Among Australia’s seven states and territories, New South Wales (NSW) has the largest prison population of 12 917 people in prison as of June 2024,^15^ accounting for over one-quarter of the Australian prison population.^15 16^ Consistent with other countries with Indigenous populations, Australia and, more specifically, NSW have a 10-fold over-representation of Indigenous Australians in custody.^15 17^ Moreover, the prison population is mainly male,^8 15^ relatively young compared with the general population^8^ and over one-third are on remand (ie, people who are yet to be tried and/or sentenced, therefore, are not serving a custodial sentence).^15^

Compared with the general population, people in NSW prisons experience a disproportionate burden of communicable and non-communicable diseases.^18^ The burden of health conditions observed in the NSW prison system includes hepatitis B, C and HIV,^1118^,^21^ as well as drug and alcohol misuse,^11 18 21^ mental and other physical health conditions.^11 18 19 21 22^ The health disparities observed in the NSW prison population compared with the general population are like others globally.^1221 23^,^25^

### The New South Wales prison system

There are 34 prisons across NSW, with three run by private operators.^26^ Corrective Services NSW (CSNSW) is the government authority that runs the 31 public prisons. CSNSW’s remit is to maintain order and security of these facilities. Alongside CSNSW, the Justice Health and Forensic Mental Health Network (Justice Health NSW) provides healthcare to people in prison. Justice Health NSW is a statutory health organisation established under the Health Services Act (NSW) 1997.^27^ Justice Health NSW provides healthcare to approximately 30 000 people annually.^28^ Services provided to people in prison include primary care, Aboriginal health, mental health, population health, oral health, women’s health and drug and alcohol programmes. However, access to these services has been observed to vary from site to site.^29^ For example, a small rural correctional centre typically does not have the same health service provision as a large metropolitan one. Moreover, primary and limited secondary healthcare services are provided akin to a community out-patients clinic, with people requiring acute care transferred to a community healthcare provider.^10^ In principle, healthcare services provided to people in prison are done so underpinned by a model of equivalence of care as stated in the United Nations Nelson Mandela Rules.^30^ Using a human rights-based perspective, where healthcare authorities should achieve equivalent health outcomes among people in prisons, not just the provision of equivalent care, when compared with the general population.^31^ A new and previously unexplored way of achieving equivalent health outcomes in the prison context is through the recently developed World Health Organization (WHO) health literacy development approach.^32^

### Health literacy of those in prison

Health literacy is recognised as a multidimensional concept,^33^,^35^ a social determinant of health^36^ and a mechanism to improve healthcare systems and health outcomes of individuals and communities.^34^ Individual health literacy represents how people access, understand, appraise, remember and use health information and services to maintain and improve their health.^34^ In contrast, community health literacy extends the concept to include the people around an individual as well as the way organisations provide healthcare along with the influence of both cultural and contextual norms.^32 34^ Thus, health literacy is not just a personal characteristic; it is also a characteristic of interactions between an individual’s social and healthcare environment. In this milieu, a person’s lack of skills or confidence can be alleviated by the resources available within their immediate context and community.

Despite the well-documented health disparities between people in prison and general populations, the health literacy of people in prison has been rarely investigated. To the authors’ knowledge, only two studies have examined health literacy using a multidimensional measure.^29 37 38^ Both studies reported that people in prison had lower health literacy, with descriptions varying depending on the measurement tool used. In their 2021 study, Mehay *et al*^38^ explored the health literacy of a group of young males (n=104; age range 18–21 years) in a single English prison using a modified version of a European health literacy survey. They reported that the majority of participants had some health literacy limitations. However, as noted by the authors,^38^ these findings cannot be generalised due to the limitations associated with the study setting and the context-specific nature of health literacy.

The second study of 471 people, undertaken within Justice Health NSW, using the nine-dimension Health Literacy Questionnaire (HLQ), reported that this population had substantially lower scores than the general Australian population.^29 37^ Comparisons between subpopulations (eg, sex and legal status) exhibited different health literacy strengths and challenges,^29^ demonstrating that the prison population is not homogenous. These HLQ data are integral to the new WHO Health Literacy Development approach that embraces variance in subpopulations and builds interventions based on the observed strengths and challenges.^32 34^ Such an approach is timely, as recent literature has highlighted that health interventions in prison environments lack critical evidence to generate and sustain improved health outcomes for people in prison.^39^ Moreover, health interventions in prison usually address areas such as mental health,^40 41^ substance use^42 43^ and infectious disease,^44^,^46^ with a small number of studies conducted in Australia and none yet to consider health literacy.

### A health literacy development approach: the Optimising Health Literacy and Access (Ophelia) process

According to the WHO, health literacy development refers to how the over-arching health system inclusive of ‘health workers, services, organisations and policy-makers (across government sectors and through cross-sectoral public policies) build the knowledge, confidence and comfort of individuals, families, groups and communities through enabling environments.’ (32, p. XIII). The Optimising Health Literacy and Access (Ophelia) process is a widely used health literacy development approach,^47 48^ which is systematic and grounded in the needs of the healthcare system end user.

Ophelia captures a population’s health literacy strengths and challenges by undertaking a needs assessment using the HLQ.^49^ The needs assessment explores how individuals’ access, understand and use health information and services available to them in a particular context. Equity is at the heart of the Ophelia process, enabling those with lived experience to specify and codesign needed and wanted solutions when accessing and using healthcare services. Further, the approach draws on intervention mapping, quality improvement collaboratives and realist synthesis to build and refine fit-for-purpose and effective interventions.^47^

**Figure 1:**
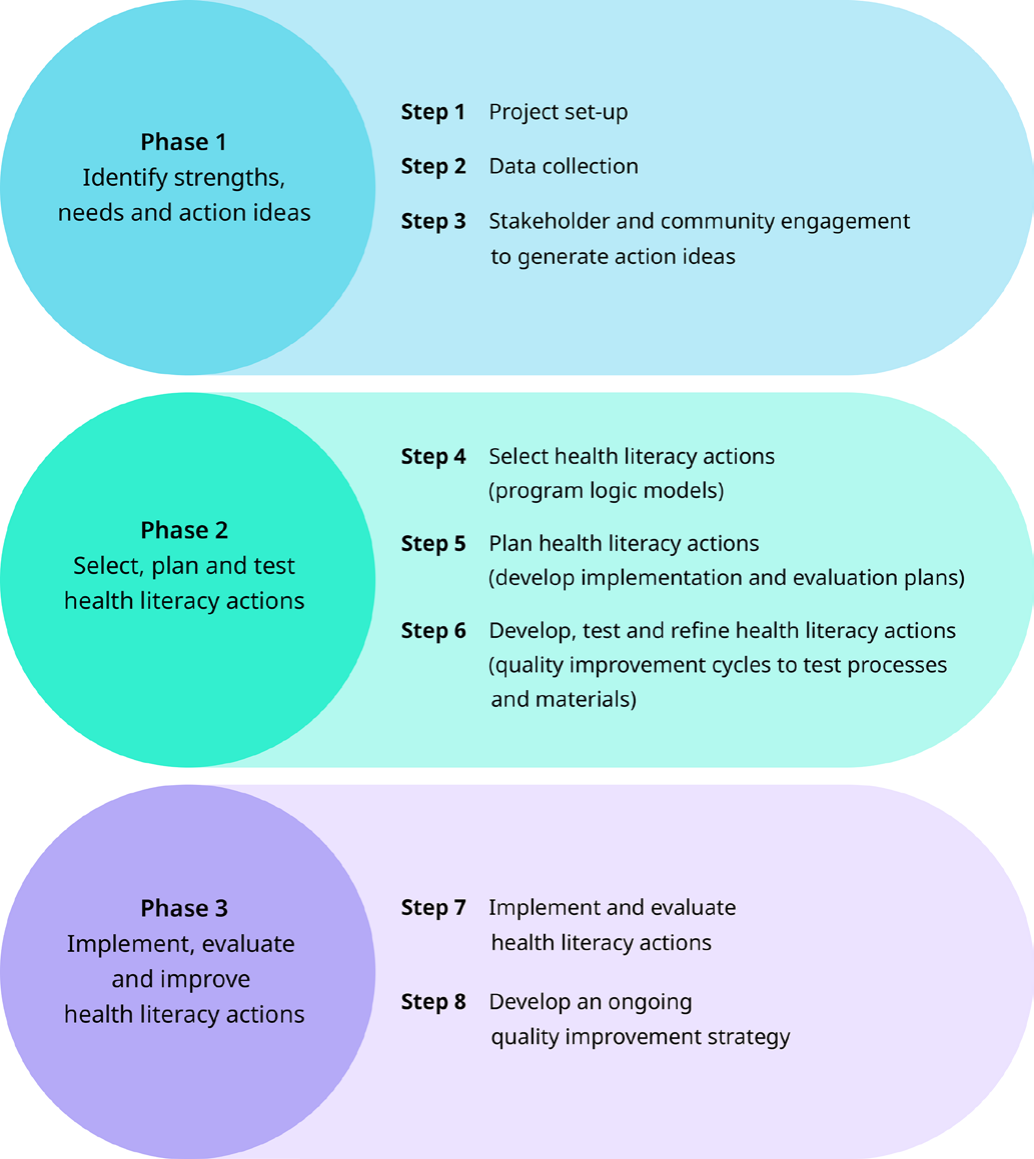
The three phases and eight steps of the Ophelia process. Source: Reproduced (with permission) from Osborne.^67^

The Ophelia process consists of three phases (figure 1), underpinned by eight guiding principles (table 1).^47 48^ Throughout each phase, close collaboration with stakeholders, including those with lived experience, frontline personnel and managers, is undertaken to guide the project and build organisational capacity.^47 48^

**Table 1:** The eight principles of the Ophelia (Optimising Health Literacy and Access) process

Principle	Description
1. Focus on outcomes	Focus on improving health and well-being outcomes
2. Driven by equity	Focus on increasing equity in health outcomes and access to services for people with varying health literacy needs
3. Driven by local wisdom	Prioritise local wisdom, culture and systems
4. Diagnosis of local needs	Respond to locally identified health literacy needs
5. Codesign approach	Engage all relevant stakeholders in the codesign and implementation of actions
6. Responsiveness	Respond to the varying and changing health literacy needs of individuals and communities
7. Applied across systems	Focus on improvement at and across all levels of health systems
8. Sustainable	Focus on achieving sustained improvements through changes to environments, practices, cultures and policies

Source: Reproduced (with permission) from Osborne.^67^

The utility of the process has been recently demonstrated through a series of WHO National Health Literacy Demonstration Projects.^32^ It has been applied in a range of countries and settings to build effective interventions, such as in disease-specific groups,^50^ cancer screening,^51^ older people,^52^ community,^53^,^55^ hospitals^56 57^ and is the primary health literacy development method being applied in an EU4Health Joint Action on Heart Disease and Diabetes programme across 24 projects in 14 countries.^58^ Previous applications of the Ophelia process have led to the development and implementation of interventions across various settings.^48^ These have included increased engagement with breast screening^51^ and reduction in hospitalisation among older people with chronic obstructive pulmonary disease.^59^ However, it has yet to be applied in a prison context where people may experience diverse challenges and vulnerabilities.

### Applying codesign methods in prisons

Central to the application of the Ophelia process is stakeholder engagement and codesign. Codesign is a form of participatory research engagement that sits on a spectrum from informing to empowering individuals and communities throughout a research project.^60^ For this protocol, we use codesign as a form of meaningful collaboration between stakeholders to solve an identified problem.^61^ The codesign process seeks to ensure end-user participation in designing a solution,^62^ with engagement ranging from relatively passive participation to highly active involvement.^63^

The prison context presents an array of structural (eg, institutional approvals, physical barriers and access) and ethical issues for careful consideration to ensure successful codesign is undertaken with people in prison.^64^ For example, in NSW, researchers must first receive ethical approval to engage people in prison in research activities,^65 66^ limiting their involvement in the conception phase of research. Further, when undertaking codesign activities in prisons, researchers must navigate complex informed consent processes, address power imbalances and build trust through culturally safe and respectful engagement.^64^ Despite these potential barriers to codesign in the prison context, the eight Ophelia process principles (table 1), combined with extensive stakeholder engagement and strong project governance, provide guidance to overcome and enable codesign activities within this challenging context.

### Research aims

The Health Literacy in Prisons Project aims to apply a health literacy development approach (the Ophelia process) to the NSW prison system to codesign actions and solutions to improve health service quality and health outcomes for people in prison. This paper aims to detail Ophelia Phase 1 methods and provide an overview of the subsequent study phases in the NSW prison context.

## Methods and analysis

### Patient and public involvement

The involvement of people in prison (eg, patients) and members of the public in protocol development and ongoing project governance is integral to this study and follows the Ophelia principles of codesign and user engagement.^47 48 67^ The involvement of stakeholders is outlined below.

### Protocol development

People in prison were not involved in the protocol development for Ophelia Phase 1. The current protocol was developed through consultations with representatives from key stakeholder organisations, including Aboriginal Community Controlled Health Organisations, Justice Health NSW and CSNSW personnel and health services decision-makers (eg, Executives). People in prison, Justice Health NSW and CSNSW personnel will be involved in generating ideas for interventions. People in prison, Justice Health NSW and CSNSW personnel, health services decision-makers and governance group members (described below) will be involved in selecting, developing and implementing interventions in Ophelia Phases 2 and 3.

### Project governance

Given the ethical considerations (eg, access, payments, informed consent, recruitment and trust) and requirements (ie, community oversight of research involving Aboriginal people) associated with conducting research with people in prison,^64^ two project advisory groups have been established to provide ongoing governance:

1. Aboriginal Community Reference Group. This group of external independent community members with experience in representing their community will provide input into the proposed research, aims, purpose and recruitment. The group will provide feedback and guidance on study activities and research findings.
2. Health Literacy Project Advisory Group. This group was established to advise and assist the investigators in achieving the project aims. The group includes representatives from across both the health and corrective services structures of NSW prisons. They will provide strategic and operational advice to maximise the practical and policy impact, advise on the interpretation of research findings, contribute to prioritisation of identified solutions and contribute to the refinement and implementation of interventions informed by health literacy (ie, health literacy-informed interventions).

Both groups will play a pivotal role in assessing research findings for implementation and scalability due to their oversight and decision-making authority within the organisations and project team. By critically evaluating the research findings, these two groups will ensure that proposed actions and solutions (ie, interventions) align with strategic objectives and are feasible for implementation within the NSW prison context. Additionally, their involvement will facilitate the identification of potential barriers to methods and the development of strategies to address them, thereby enhancing the likelihood of successful adoption and dissemination of health literacy-informed interventions. Overall, their assessment helps to bridge the gap between research and practice, ensuring that evidence-informed solutions can be effectively implemented to maximise their impact. Moreover, the research team will partner with both governance groups to ensure the eight guiding principles of the Ophelia process (table 1) are applied throughout the project’s phases.

### Study design

This is a mixed-methods action research study, with the outcomes of each phase informing the next.

### Phase 1: identification of local strengths, challenges and issues

Phase 1 of the Ophelia process^67^ uses a mixed-methods sequential explanatory design. The first step is to set up a project team to define the scope and aim of the project. A health literacy needs assessment of intended beneficiaries (ie, people in prison) will be undertaken to obtain detailed information about their health literacy strengths and challenges using the HLQ.^67^ The HLQ is a 44-item questionnaire that assesses nine independent yet complementary scales (with four to six items per scale).^49^ The nine HLQ scales broadly cover concepts related to how people engage with and use health services and information and their social support (see table 2 and online supplemental file 1 for a description of each scale). A total score is not generated for the HLQ, with each scale score calculated by the sum of responses divided by the number of items in the scale and reported separately.

**Table 2:** Health Literacy Questionnaire scales^49^

Scales	Number of items
*Scales 1–5 are rated on a 4-point agreement scale (1: strongly disagree to 4: strongly agree*)	
1. Feeling understood and supported by healthcare providers	4
2. Having sufficient information to manage my health	4
3. Actively managing my health	5
4. Social support for health	5
5. Appraisal of health information	5
*Scales 6–9 are rated on a 5-point ease scale (1: cannot do or always difficult to 5: always easy*)	
6. Ability to actively engage with healthcare providers	5
7. Navigating the healthcare system	6
8. Ability to find good health information	5
9. Understanding health information well enough to know what to do	5

Ophelia Phase 1 for the current study will use existing HLQ and participant characteristic data (n=471) collected as part of the Justice Health NSW 2021 Health Literacy Study (described above), which has been reported elsewhere.^29 37^ The previously collected HLQ data^68^ will be analysed using cluster analysis. Cluster analysis, a multivariate technique, will be used to uncover health literacy profiles among groups of individuals with similar patterns of health literacy scores across the 9 HLQ scales.^68^ This method will provide a more nuanced understanding of the health literacy strengths and challenges experienced by people in prison and inform the development of vignettes (see below). Semi-structured interviews will be undertaken with people in prison to gain further insights into how people engage with health information and services. A series of vignettes, one per cluster, will then be generated from the cluster analysis and typically reveal patterns of health literacy strengths and challenges. The vignette development is also informed by the demographic profile of a cluster and from (deidentified) narratives from the semi-structured interviews.

Vignettes will be codesigned with each of the stakeholder groups. The vignettes are typically incrementally improved through rounds of review with stakeholders, including people in prison, corrective and health staff and members of the two governance groups. Once finalised, vignettes will be presented at a series of ideas generation workshops with 6 to 12 participants. In separate workshops, people in prison and personnel (health and corrections) will discuss several vignettes and suggest ideas and solutions to help the issues raised for the persona depicted in each vignette.

### Primary data collection

#### Semi-structured interviews

To inform vignette development, semi-structured interviews will be conducted with people in prison to gather contextual data about their experiences of healthcare while in prison, such as privacy, demographic characteristics, and explore why they scored low or high on particular HLQ scales (online supplemental file 2). Using purposive recruitment,^69^ people in prison with varying health literacy scores will be invited to participate in the interviews. It is anticipated that up to 10 interviews will provide sufficient contextual data to support vignette development. Interviews will be conducted face-to-face and audio recorded with participants’ consent. The interviews are expected to take 30 min to complete. Semi-structured interview data will be transcribed. These data will then be analysed to extract valuable contextual insights to inform the development of the vignettes.

#### Ideas generation workshops and Yarning circles

Vignettes developed from the Phase 1 needs assessment and interviews will be presented to different stakeholder groups (ie, people in prison, Justice Health NSW and CSNSW personnel) in ideas generation workshops and yarning circles (ie, a conversational form of Aboriginal data collection where researchers listen ‘to participants’ stories about their lived experiences, feelings, thoughts and ideas on the research topic’^70^ (p38)) to generate health literacy-informed interventions to improve health service quality and health outcomes for people in prison.

Workshops and yarning circles will be about 1 hour to 2.5 hours, depending on the stakeholder group. Six to ten ideas generation workshops and yarning circles will be undertaken with participants across NSW prisons and Justice Health NSW sites, with between 6 and 12 participants per group. A group size of 6 to 12 participants has been chosen based on evidence to enhance participants’ comfort and active participation.^71^ Each group will contain participants from that stakeholder group. Depending on the stakeholder group, participants will be recruited via various direct and indirect approaches. For example, people in prison will be recruited by promoting the study via Inmate Development Committee meetings (ie, a prison community meeting), study posters and direct recruitment in the prisons. Justice Health NSW and CSNSW personnel will be recruited by sharing study information via email, intranet pages, internal communications and study posters in work locations. The goal is to recruit 30 people in prison, 30 Justice Health NSW and 20 CSNSW personnel to participate in ideas generation workshops and yarning circles. This goal has been set as it will provide a cross-sectional representation of the different stakeholder groups in NSW prisons. Ideas generation workshops and yarning circles will continue until data saturation. The stakeholder inclusion and exclusion criteria for ideas generation workshops and yarning circles are outlined in table 3.

**Table 3:** Stakeholder group inclusion and exclusion criteria for ideas generation workshops and yarning circles

Stakeholder group	Inclusion criteria	Exclusion criteria
Justice Health NSW personnel	Participate voluntarily. Able to provide written informed consent. Have approval from the supervisor or manager, if required, to participate.	They are not currently an employee of Justice Health NSW. Do not have supervisor or manager permission, if required.
CSNSW personnel	CSNSW personnel and managers who have or had a role in the provision of supervision for adults in prison. Participate voluntarily. Able to provide written informed consent. Have approval from the supervisor or manager, if required, to participate.	They are not currently an employee of CSNSW. Do not have supervisor or manager permission, if required.
People in prison	A person aged 18 years or older. Currently in the care of CSNSW and Justice Health NSW. Participate voluntarily. Able to comprehend English. Able to provide written informed consent.	They are under the age of 18 years. They do not speak English or are unable to comprehend English. They are not able to provide written informed consent.

Following informed consent procedures, the facilitators will provide an overview of the project and the aims and process of the ideas generation workshop or yarning circle. A facilitator will then read a vignette to the group and start the discussion by asking the first guiding question. Four questions will guide the ideas generation workshops for Justice Health NSW and CSNSW personnel, while separate workshops and yarning circles, guided by a different set of four questions, will be conducted with people in prison (table 4). These questions have been designed to encourage participants to relate to the lived experience expressed in each vignette and emote genuine engagement.^54 67^ Throughout the ideas generation workshop or yarning circle, the facilitators will encourage participants to expand and build on the actions and solution ideas generated. Following the discussion of each vignette, facilitators will summarise the discussion with participants to confirm that the action and solution ideas are understood. This process will be repeated for subsequent vignettes. Three to five vignettes are expected to be discussed in each idea generation workshop or yarning circle.

**Table 4:** Stakeholder group question routes for ideas generation workshops and yarning circles

Stakeholder group	Question route
Justice Health NSW and CSNSW personnel	Do you see people like this person in your centre? What are the issues this person is dealing with? What strategies could be used to help this person? If there were lots of people like this person, what could our organisations do to improve outcomes for these people?
People in prison	Do you think there are people like this around you in prison? What are the main issues this person is dealing with? What do you think could be done to improve care for this person? What could our organisations do to help people like this person in prison?

### Data analysis

#### Cluster analysis

Descriptive analysis will be conducted on the 471 survey responses, including participant characteristics and HLQ scale scores using SPSS V. 29.0.^72^ To generate health literacy profiles, hierarchical cluster analysis following Ward’s method^73 74^ will be undertaken as recommended by the Ophelia Manual^67^ and previous Ophelia studies.^50^,^5355^ Currently, no consensus exists for an adequate sample size to undertake cluster analysis.^75^ However, based on previous Ophelia studies, Hawkins *et al*^50^ suggest that sample sizes over 100 can generate a robust cluster solution and provide rich information about potential subgroups in a population. Therefore, the 471 survey responses previously collected are assumed sufficient for this study.

A cluster solution of up to 20 clusters will be generated, and the patterns of health literacy profiles will be explored alongside the corresponding participant demographic characteristics. A separate cluster solution will be examined for individuals who identified as Aboriginal, Torres Strait Islander or both. The separate cluster solution for Aboriginal identity will be undertaken due to the over-representation of Aboriginal people in the prison system and in recognition of their cultural perspectives of health (eg, holistic view^76^) that may differ from that of the dominant Western health system in prisons. The cluster selection process will be undertaken by one researcher followed by discussions with the research team.

#### Ideas generation workshops and Yarning circles

Health literacy action and solution ideas identified through the workshops and yarning circles will undergo thematic analysis via an inductive theoretical approach as recommended in the Ophelia Manual.^67^ Actions identified to improve health literacy are expected to broadly fall within three contexts,^47^ that is, with individuals, health service providers and organisational and policy structures. The analysis will be undertaken by one researcher and reviewed by other research team members.

Following completion of the thematic analysis of the identified local health literacy actions and solutions, a prioritisation workshop will be held with key stakeholders including members of both project advisory groups, Justice Health NSW and CSNSW personnel and health decision-makers. The actions and solutions selected to be implemented and evaluated in Phase 3 will be determined by the outcome of Phase 2 activities.

### Phase 2: select, plan and test health literacy actions

In Phase 2 of the Ophelia process,^67^ local stakeholders—including advisory groups, researchers and other relevant stakeholders—will review and become familiar with the ideas generated during Phase 1. This familiarisation will take place through workshops focused on discussing the depth and breadth of the ideas, as well as identifying patterns, linkages and synergies. Stakeholders will then evaluate these ideas based on local priorities and the project’s objectives. Additionally, a rapid literature review will be conducted to determine whether evidence exists to support the identified and prioritised actions.

A programme logic model (ie, a model outlining how a programme is intended to function by describing the causal links and mechanisms required to achieve the desired outcome^77^) is then developed to align interventions with project objectives and guide implementation. Once developed, intervention and evaluation planning will ensue. This will involve identifying the implementation team, defining roles and responsibilities and confirming timelines and budgets. The final step in Phase 2 will be to develop, test and refine the health literacy-informed interventions^48^ using plan-do-study-act cycles—structured, iterative testing of a potential intervention where each cycle incrementally improves the product.^78^

### Phase 3: implement, evaluate and improve health literacy actions

The final phase, Phase 3 of the Ophelia process,^67^ is to implement and evaluate the selected health literacy interventions. This phase uses the finalised products derived from the preceding phases, that is, ready to be implemented interventions. Feedback will be collected from implementation sites through process and outcome evaluations. The project team will also develop an ongoing quality improvement strategy to ensure the sustained effectiveness of the interventions.

## Discussion

The needs of people in prison have been widely detailed in literature over the past three decades; however, only recently have the health literacy strengths and challenges been investigated and described.^29 37^ To date, limited studies have systematically used health literacy to develop localised actions and solutions to reduce health disparities observed in prison populations.

Healthcare authorities and organisations providing care to people in prison need to explore new ways of working to reduce the health disparities observed in this population. There needs to be a shift from the principle of equivalence of care^30^ (ie, equality in care) to a principle of equivalent health outcomes (ie, equity in health)^31^ to help reduce these disparities. This protocol should generate local actions and solutions that the system end user (ie, people in prison) needs and wants while meeting environmental constraints.

The Ophelia process systematically generates such evidence with a focus on developing localised practice-based solutions. While the Ophelia process draws on insights from other intervention development approaches, including Intervention Mapping,^79^ Quality Improvement Collaboratives,^80 81^ realist synthesis,^82^ and the Medical Research Council (MRC) framework for developing and evaluating complex interventions,^83^,^85^ it seeks to ensure the inputs are based on deep understanding of individual health literacy needs in context (ie, through the HLQ). Ophelia also employs ideas generation workshops facilitated by empirically derived vignettes, including vignettes of people who may be experiencing substantial marginalisation and vulnerability, with the embedded health literacy challenges. The ideas generation workshops ensure both people in prison and professionals have equal voice on what solutions may improve health and service access in highly localised contexts. Consequently, the process focuses on both bottom-up and top-down codesign approaches, embedding the lived experience of people in prison and organisational personnel who hold solutions to provide care within a heavily scrutinised and rigid system. This protocol details an adaptation to the Ophelia process for the NSW prison context, which is highly relevant to other prison and healthcare authorities in Australia and globally looking to meet the needs of those in their care.

Potential implications relevant to other prison and healthcare authorities from the Health Literacy in Prisons Project are that stakeholders with lived experience, importantly, will have their voices heard and that this can influence policy and practice to improve how this voice of tough-to-reach individuals is included in service delivery and planning. This process will also inform how Justice Health NSW and CSNSW (and other Australian and international prison and healthcare authorities providing care to incarcerated populations) provide the types of services and information people in prison need and want to manage their health. Furthermore, the Ophelia process could be used by other services providing care to people in prison to understand the strengths and challenges of the people they care for. Understanding and improving the health literacy of people in prison has the potential to reduce observed health disparities and inequities in a population that experiences substantial marginalisation, moving prison healthcare authorities to aim for equivalent health outcomes, not just equivalence of care.

### Potential limitations of this study

Potential limitations of the current protocol include those who did not speak English, did not access the health clinic or were in rural or remote prisons were excluded from the initial health literacy needs assessment.^29 37^ Therefore, the needs assessment may not fully represent the voice and lived experience of people in NSW prisons. The study will aim to mitigate this limitation by seeking advice from the two project advisory groups. Moreover, the voices of people with similar experiences may again be excluded in the ideas generation workshops. Where opportunities present to include this voice and in line with codesign principles, modifications will be made to ensure the lived experience of these groups is heard and included in data collection activities. The study setting poses several potential limitations, such as conducting codesign, engaging actively with stakeholders and developing feasible interventions within a restrictive environment. To address these challenges, a strong governance structure has been established to minimise impacts, mitigate perceived barriers and enhance engagement with study activities. This structure aims to foster local ownership and ensure that all stakeholder voices are heard and treated equally.

## Ethics and dissemination

Ethical approval to undertake Phase 1 of the Ophelia process has been granted from the following Human Research Ethics Committees: Swinburne University of Technology (Ref: 20236977–15461), Justice Health NSW (Ref: 2022/ETH01433), Aboriginal Health and Medical Research Council (Ref: 2007/22) and the Corrective Services Ethics Committee (Ref: D2022/1452326). Dissemination of the study findings will be the Justice Health NSW codesign process and ownership of the project through authentic engagement with people with lived experience and health and corrective staff. It will also be disseminated through publication in a PhD thesis, peer-reviewed research papers and conference presentations. Written informed consent will be obtained from all participants. In accordance with Chapter 2.3 of the National Statement on Ethical Conduct in Human Research,^65^ a waiver of consent has been granted to access and undertake secondary analysis of the previously collected HLQ data.

## supplementary material

10.1136/bmjopen-2024-092128 online supplemental file 1

10.1136/bmjopen-2024-092128 online supplemental file 2
