# Supplementary material for: Applying co-design health literacy development in Australian prisons: protocol for system-wide application of the Optimising Health Literacy and Access (Ophelia) process
Source: BMJ Open. 2025 Apr 7;15(4):e092128. doi: 10.1136/bmjopen-2024-092128 (PMC11977488; doi:10.1136/bmjopen-2024-092128)
Supplement: online supplemental file 2 [file bmjopen-15-4-s002.DOC]

**Supplementary File 2 – Semi-structured interview questions and guide**

| **Access & Use** | |
| --- | --- |
| Questions | |
| 1 | Do you access health services in prison?  If yes, how do you access the health services?  If not, why do you not access health services? |
| 2 | Do you access health information (i.e. pamphlets, fact sheets) when in prison?  If yes, how do you access the health information?  If not, is there anything stopping you from accessing health information? |
| 3 | How often do you access the health services in prison?  Would you like to have more access to the health services?  How can we do to help your access? |
| 4 | When you accessing the health care, who do you normally see?   - Nurse - Doctor - Dentist - Physiotherapist etc. |
| **Barriers to health care** | |
| 1 | Do you have any barriers to accessing the health care you need in prison?  If yes, what are these barriers?  If not, is there anything that could be done to improve your access? |
| 2 | You mentioned *[XXXXX]* as a barrier, can you tell me more about that? |
| **Connecting to healthcare providers** | |
| 1 | How do you speak with healthcare providers (i.e. nurses, doctors)?  Do you have any suggestions on how this could be easier? |
| 2 | When speaking with a healthcare provider, do you have any problems understanding them?  If yes, what are the issues you are having?  If not, what allows you to understand them? |
| **Privacy** | |
| 1 | Do you have any concerns over the privacy when accessing healthcare in prison? |
| 2 | Are you confident that your health information is in safe hands? |
| **Support** | |
| 1 | Do you have any family or friends who can help you when accessing healthcare when you need to? |
| 2 | How often do you have access to the people who can help you? |
| **Further questions may be asked in relation to the barriers (low scores) they have as identified in their questionnaire. “From your Questionnaire results, you seem to have difficulty with *[XX]* – can you explain what you mean by this?”** | |
| **Scale 1: Feeling understood and supported by healthcare providers** | |
| **Scale 2: Having sufficient information to manage my health** | |
| **Scale 3: Actively managing my health** | |
| **Scale 4: Social support for health** | |
| **Scale 5: Appraisal of health information** | |
| **Scale 6: Ability to actively engage with healthcare providers** | |
| **Scale 7: Navigating the healthcare system** | |
| **Scale 8: Ability to find good health information** | |
| **Scale 9: Understand health information well enough to know what to do** | |

**Further Questions**

| **HLQ Scale** | **Higher HLQ Score** | **Lower HLQ Score** |
| --- | --- | --- |
| **1. Feeling understood and supported by healthcare providers** | Do you feel understood and supported by your healthcare providers? Why do you feel this way? | Do you feel understood and supported by healthcare providers? Has it always been this way? What would help to improve these relationships? |
| **2. Having sufficient information to manage my health** | What helps you to feel you have enough information to manage your health and make decisions? | What would help you to feel confident that you have enough information about your health? |
| **3. Actively managing my health** | How did you learn to manage your health? What do you do to manage your health? | What do you do to manage your health? What do you need to help you manage your health differently? |
| **4. Social support for health** | What kind of help do you get from your family, friends or community for your health? | What kind of help do you get from your family, friends or community for your health? |
| **5. Appraisal of health information** | What helps you to understand health information? How do you work out what is best for you? | What would help you to understand health information and work out what is best for you? |
| **6. Ability to actively engage with healthcare providers** | How comfortable do you feel about talking about your health with healthcare providers? What has helped you with this? | How comfortable do you feel about talking with healthcare providers? What would help you to feel more comfortable to talk with them about your health? |
| **7. Navigating the healthcare system** | How do you find out about the health and support services that you need? | What would help you to find out about health and support services that you need? |
| **8. Ability to find good health information** | What has helped you to find information about your health? | What would help you to find information about your health? |
| **9. Understand health information well enough to know what to do** | What has helped you to understand written health information? | What would help you to understand written information about your health? |
