# Supplementary material for: Applying co-design health literacy development in Australian prisons: protocol for system-wide application of the Optimising Health Literacy and Access (Ophelia) process
Source: BMJ Open. 2025 Apr 7;15(4):e092128. doi: 10.1136/bmjopen-2024-092128 (PMC11977488; doi:10.1136/bmjopen-2024-092128)
Supplement: Supplementary file 3 [file bmjopen-2024-092128.reviewer_comments.pdf]

PEER REVIEW HISTORY

BMJ Open publishes all reviews undertaken for accepted manuscripts. Reviewers are asked to complete a checklist review form and are provided with free text boxes to elaborate on their assessment. These free text comments are reproduced below.

ARTICLE DETAILS

Title (Provisional)

Applying a co-design health literacy development approach for diverse health services in Australian prisons: A protocol for the Optimising Health Literacy and Access (Ophelia) process

Authors

Gill, Scott W; Bowman, Julia; Cheng, Christina; Shaw, Caron; Hampton, Stephen; Hoey, Wendy; Osborne, Richard H

VERSION 1 - REVIEW

**Reviewer**	**1**
**Name**	**Mehay, Anita**
**Affiliation**	**City St George's University of London**
**Date**	**27-Aug-2024**
**COI**	**None**

Thank you for the opportunity to review this protocol paper about an interesting project looking to use a well-known approach to co-design actions and solutions relating to the health of people in prison. I commend the research team for their focus and efforts in this important context and population group.

Overall, there are some strengths of the research including the application of an existing Ophelia process to a new context and population group with engagement of a large number of prisons. This reflects a potentially exciting collaboration and any findings from the research (if conducted well and successfully) will likely be important and add to the existing evidence base.

However, there are also some significant areas requiring further clarification which I highlight:

- Prisoner health is as much determined by the prison environment itself as well as healthcare provision. There is little to no reference to the evidence base on what is known already about the prison context and how they are inherently unhealthy places which present huge challenges to developing and delivering any health interventions. For instance, the structural barriers, impact of the complex social work of prisoners, high levels of violence and overcrowding, detachment from friends and family, etc. etc. How can (if at all) the Ophelia process take this context into account?

- Co-design seems to be a core element of the Ophelia process but this is not well described or conceptualised. There are various interpretations of co-design (including its use with other terms such as

co-production, co-creation etc.) so the terminology needs more clarification in this paper. This is particularly important as prisons are largely designed to disempower, control and strip people of their agency. A comprehensive and thoughtful outline of how co-design applies within this context would be critical in this paper (which might include the parameters and limits of the process in prisons).

- The role and involvement of people in prison in this research also lacks sufficient detail and explanation. The co-design element and the discussion implies that the prisoner voice is centred in the research, but this does not come through in the methods which suggest that prisoners are involved in providing data from the HLQ and in some engagement in workshops to generate ideas. There needs more description and analysis of the co-design element (as suggested in the previous point) with a clear sense of how this applies in prisons, how prisoners are/are not involved in this and why, and what this means overall.

- Prisons contain a highly vulnerable group which includes a disproportionate representation of people from indigenous and ethnic minority groups. The authors do recognise this but there is no further explanation of why this disparity/injustice exists and why/how the Ophelia process is particularly useful and relevant (i.e. the codesign element again). I can see there will be an advisory group which is embedded but it is also not clear how these will be embedded and how mitigate against the multiple power imbalances which exist with both prisons, academic researchers and other professional stakeholder groups.

- Although I appreciate that it is difficult to outline what the intervention(s) might look like considering the use of the co-design/Ophelia process, the paper would benefit with providing some examples and principles, values and initial ideas/parameters that will guide the co-design process. For example, is this about behaviour change interventions through leaflets/posters or healthcare redesign or something else? I assume there will be interventions which are just not possible in the prisons (or across all the prisons). Also, how does the Ophelia process link with (or not) existing frameworks to guide intervention design (including the MRC framework for developing and evaluating complex interventions)?

- The research aims to apply the Ophelia approach to improve health service quality and health outcomes. There is a useful outline of the Ophelia process and the application in a range of countries and settings to build effective interventions. It would be useful to outline the research around the development of any actual and relevant interventions and their effectiveness in improving outcomes (if this exists). There is also little mention what key outcomes of interest are in this study or if this will be clarified as part of the co-design process.

- There are multiple ethical issues when conducting research in prison, including how they will gain informed consent, issues around power differences in prison, building trust for true co-design etc. This is currently missing and would strengthen the protocol.

I also outline some further comments for the authors to consider:

Background:

- It would be useful to set the scene with a global focus with a sense of the prison population in Australia. Authors also offer some useful size of the prison population, and it would be useful to understand this as a rate with any meaningful comparison to other states or countries.

- Line 80: useful to add a reference to the first sentence.

- Line 80: it is not usual to describe people as having ‘experienced prison’ – it is also an ambiguous statement as not clear if the 42,000 figure refers to the prison population size currently or about people who have been in prison that year (as ‘experienced’ is past tense). Would be useful to rephrase.

- Line 87: do people in NSW prisons experience a disproportionate burden compared with other prisons in other states? Or compared with the general population? Worth clarifying this sentence.

- Line 92: how will it inevitably impact the overall health of the general population?

- Line 95: ‘People in NSW prisons are incarcerated in 34 prisons…’ – a bit of a circular sentence (the sentence is like saying - people in prison are in prison in prisons). Worth rerevising to something like ‘There are 34 prisons in NSW state …’

- Line 103: ‘However, access to these services has been observed to vary from site to site.’ - this sentence needs more information and a reference

- Lines 120-132: you state there have been two studies examining health literacy in prison – but only describe one. Would be more comprehensive to describe both considering they are the only two.

Methods and analysis

- Lines 171-180: Two advisory groups have been established ‘due to the ethical considerations and requirements …’. But it is not clear what these ethical considerations and requirements are. How will you ensure these groups are supported and meaningful co-design and participation is reached (relates to my more substantial points too).

- Lines 186: Although Table 2 is useful, it would be helpful to the reader to have a sentence or two outlining the HLQ briefly rather than having to engage with a whole table.

- Lines 187-189: although the HLQ data has been collected and reported elsewhere, it seems important to give a summary of this and rationale for re-analysing in this study.

- Lines 190-197: it is not clear who are the community members? Who will be involved in developing the series of vignettes? How will vignettes be developed? Much of this information is in the primary data collection section as well – so there is also some repetition.

- Line 201: Who are the other stakeholders?

- Lines 206-211: need to outline a bit more about a logic model and who/how it will be developed; what is the PDSA cycle and why – need a reference.

- Lines 224-225: 10 interviews lasting just 30mins with prisoners seems very short to provide the in-depth basis for vignettes etc. This needs further justification.

- Line 255: why will there be a separate cluster for individuals who identify as Aboriginal, Torres Strait Islander or both?

Discussion

- Potential limitations section – there are several limitations missing, including those relating to my more substantial points (i.e. limits of co-design in prison, extent of interventions which are feasible in prison, lack of focus on outcomes…)

Abstract

- This would require revisions in line with the comments made already.

**Reviewer**	**2**
**Name**	**Wayland, Sarah**
**Affiliation**	**University of Technology Sydney**
**Date**	**29-Aug-2024**
**COI**	**I note that I have previously recieved funding to collaborate with one of the authors (Bowman) however we do not have any current collaborations.**

Thank you for the opportunity to review this protocol. The care and attention taken to ensure that language does not focus on deficits, but the strengths of the aims of this project, are well constructured. The purpose, approach and potential outcomes of this study are clearly focu

---

**Reviewer**	**3**
**Name**	**Lander, Jonas**
**Affiliation**	**Hannover Medical School, Institute for Epidemiology, Social Medicine and Health Systems Research**
**Date**	**25-Oct-2024**
**COI**	**none**

Dear Editor and authors,

many thanks for inviting me to review this protocol, which I think addresses a timely issue in the context of HL and prisons. Please see my individual comments attached as a file, I wouldnt say this is necessarily a minor or major revision, but a revision in any case. Good luck with finalising the text and kind regards!

Applying a co-design health literacy development approach for diverse health services in Australian prisons: A protocol for Optimising Health Literacy and Access (Ophelia) process

Title:

- I find the second part of the title as of “A protocol for..” a bit confusing and it misses a “the”, doesn’t it? I understand you want to include Ophelia in the title, but at the same time its quite long then. Usually would only say “a study protocol” or sth similar – it also doesn’t say what you intend to optimize for Ophelia.

Abstract

- What are HL-informed interventions, is that different from HL-interventions?
- Can it be assumed in the abstract that any reader knows Ophelia?
- Whats the difference between needed and wanted?

Introduction

- Can you please clarify…
- Whether, in the prison context, you refer to individual HL, organizational HL or a mix of both?
- How HL is actually intended to / may be supported in the prison context, e.g. do imprisoned people have access to health info?
- Whether prisons have an interest and or obligation to work towards improving HL or related concepts?
- Examples for previous health interventions for imprisoned people (in AUS/NSW) and to what extent these worked well or not?

Methods

- What does "consultation" mean in concrete for the protocol dev?
- Its not clear if the HL needs assessment with n=471 has already been done in 2021?
- Who are community members?
- I wonder if the study design section could be adjusted, it partly reads like you explain the entire study here, and at the same time it may not give a more general impression of what kind of study this is, the theoretical approach underlying this study, etc. I understand you want to say "this is an Ophelia study" but then why wouldn't you use the last part from the introduction to outline the study design?
- I am unclear about the aspects as of program logic model and implementation and evaluation of HL actions. If I'm not mistaken, you do not provide any details on how you will operationalize these steps because the methods section ends with data analysis.
- I cant find details on how you approach (recruit) study participants for the various phases, particularly the interviews and workshop and here, imprisoned people – how do you approach them and what kind of participation do you expect?
- Workshops: can you be more concrete on how you "generate ideas"?
- Have you explained somewhere in the protocol what yarning cycles are?
- Limitations: your intended study implies quite some active participation of the various target groups, right? If so, do you not expect any challenges with really achieving this active participation?

VERSION 1 - AUTHOR RESPONSE

Reviewer: 1

Reviewers comments: Thank you for the opportunity to review this protocol paper about an interesting project looking to use a well-known approach to co-design actions and solutions relating to the health of people in prison. I commend the research team for their focus and efforts in this important context and population group.

Overall, there are some strengths of the research including the application of an existing Ophelia process to a new context and population group with engagement of a large number of prisons. This reflects a potentially exciting collaboration and any findings from the research (if conducted well and successfully) will likely be important and add to the existing evidence base.

However, there are also some significant areas requiring further clarification which I highlight:

Author response: We thank the reviewer for their comments.

Reviewers comment: Prisoner health is as much determined by the prison environment itself as well as healthcare provision. There is little to no reference to the evidence base on what is known already about the prison context and how they are inherently unhealthy places which present huge challenges to developing and delivering any health interventions. For instance, the structural barriers, impact of the complex social work of prisoners, high levels of violence and overcrowding, detachment from friends and family, etc. etc. How can (if at all) the Ophelia process take this context into account?

Author response: We thank the reviewer for their insights. We agree that prisons can be unhealthy environments. We have added multiple sections to the manuscript that address this comment; this

includes an introductory paragraph to prisons (Page 5, Line 93-101), and strengthened our governance section (Pages 11&12, Lines 233-274). Through its three phases, guiding principles, and focus on harnessing local knowledge, we believe the Ophelia process will enable the prison environment's structural and contextual components to be considered.

We acknowledge that context can greatly affect health. Given that the process is co-design in the context of prisoners' daily lives, the potential solutions relevant to prisoner's daily lives are being generated and therefore account for and embrace this complexity. We agree that examining the number, type and role the prison context(s) may have on prisoner wellbeing, however the focus of this study is to directly intervene at the prisoner and prison environment levels to achieve meaningful outcomes.

Reviewers comment: Co-design seems to be a core element of the Ophelia process but this is not well described or conceptualised. There are various interpretations of co-design (including its use with other terms such as co-production, co-creation etc.) so the terminology needs more clarification in this paper. This is particularly important as prisons are largely designed to disempower, control and strip people of their agency. A comprehensive and thoughtful outline of how co-design applies within this context would be critical in this paper (which might include the parameters and limits of the process in prisons).

Author response: We thank the reviewer for their comment. We have now added a section (Page 10, Line 207-226) in the introduction to define co-design and associated activities. Further in this section, we introduce co-design in prisons and highlight some issues within the prison context. Moreover, we have significantly strengthened the 'Patient and Involvement Section', which details our protocol development and governance structure (Pages 11&12, Lines 233-274).

Reviewers comment: The role and involvement of people in prison in this research also lacks sufficient detail and explanation. The co-design element and the discussion implies that the prisoner voice is centred in the research, but this does not come through in the methods which suggest that prisoners are involved in providing data from the HLQ and in some engagement in workshops to generate ideas. There needs more description and analysis of the co-design element (as suggested in the previous point) with a clear sense of how this applies in prisons, how prisoners are/are not involved in this and why, and what this means overall.

Author response: We thank the reviewer for their thoughtful comment. We believe we have addressed this comment in the previous two comments. Below, we have outlined some planned and potential future co-design activities for the study that enable the voices of stakeholder groups to be heard.

Stakeholder group	Activities
People in prison	1. Declaration of their health literacy challenges through completion of a health literacy needs assessment (ie the HLQ) to inform vignette development.<br>2. Participation in qualitative interviews to share their story of what it is really like to be in prison and try to manage their own health.<br>3. Review of the draft vignettes. These vignettes depict typical people in prison with a range of health literacy, health, access and sense of empowerment issues.

	4. Participation in Ideas Generation Workshops or Yarning Circle, where people in prison provide direct input and insights into what and how health and equity can be generated in their own daily context. Authentic engagement is ensured by creating confidential and psychologically safe settings for the group participation. These ideas are given equal (or higher) weight that ideas arising from staff.<br>5. Presentation back to people in prison for them to declare what they think will be useful and have meaningful impact on their wellbeing.<br>6. Participation in conversations and other relevant study activities to develop, implement, iteratively improve and evaluate health literacy-informed interventions in the prison context.
Corrective and health staff	1. Involvement in the project advisory groups, which has included providing guidance on activities related to the study and will continue and is detailed in the revised manuscript for other activities.<br>2. Review of the draft vignettes. These vignettes depict typical people in prison with a range of health literacy, health, access and sense of empowerment issues.<br>3. Participation in Ideas Generation Workshops, where staff provide direct input and insights into what and how health and equity can be generated in their own daily context. Authentic engagement is ensured by creating confidential and psychologically safe settings for the group participation. These ideas are given equal (or higher) weight that ideas arising from people in prison.<br>4. The planned validation and prioritisation of action ideas generated from the Ideas Generation Workshops.<br>5. Participation in a prioritisation workshop that will inform the next phases of the Health Literacy in Prisons project.<br>6. Involvement in meaningful conversations to help select, develop, implement, iteratively improve and evaluate health literacy-informed interventions in the prison context.

Reviewers comment: Prisons contain a highly vulnerable group which includes a disproportionate representation of people from indigenous and ethnic minority groups. The authors do recognise this but there is no further explanation of why this disparity/injustice exists and why/how the Ophelia process is particularly useful and relevant (i.e. the codesign element again). I can see there will be an advisory group which is embedded but it is also not clear how these will be embedded and how mitigate against the multiple power imbalances which exist with both prisons, academic researchers and other professional stakeholder groups.

Author response: We thank the reviewer for their comment. This protocol is not seeking to describe and evaluate why the disparities exist. We believe we have addressed the issues around the Ophelia co-design elements in the previous three comments.

Reviewers comment: Although I appreciate that it is difficult to outline what the intervention(s) might look like considering the use of the co-design/Ophelia process, the paper would benefit with providing some examples and principles, values and initial ideas/parameters that will guide the co-design process. For example, is this about behaviour change interventions through leaflets/posters or healthcare redesign or something else? I assume there will be interventions which are just not possible in the prisons (or across all the prisons). Also, how does the Ophelia process link with (or not) existing frameworks to guide intervention design (including the MRC framework for developing and evaluating complex interventions)?

Author response: We thank the reviewer for their comment. We agree that the nature of co-design means that we should not speculate on what intervention(s) participants will suggest, and we understand that not all suggestions will be implementable and scalable.

As stated in the Ophelia process manual, intervention ideas arising from the Ideas Generation Workshops, may be organised into interventions across 4 levels (individual, community, practitioner or policy) or they may be organized into more relevant levels depending on the context. How we intend these ideas may be organised in the NSW prison context is described on page 20, Lines 410-415.

The Ophelia process is based on three primary theoretical approaches: Intervention mapping, Quality Improvement Collaboratives and realist synthesis. This is made clear when we first introduce the concepts of the Ophelia process (Page 8, Lines 187-189). We have further discussed this and made reference to the MRC framework in the Discussion (see page 21 & 22, lines 456-465).

The principles and governance structure that will guide the research team have been outlined in the manuscript in Table 1 (Page 9, Line 197-198) and Pages 11&12, Lines 233-274, respectively. Further, to clarify, the initial parameters are guided by what the stakeholders engaged want and what they prioritise throughout applying the Ophelia process.

Reviewers comment: The research aims to apply the Ophelia approach to improve health service quality and health outcomes. There is a useful outline of the Ophelia process and the application in a range of countries and settings to build effective interventions. It would be useful to outline the research around the development of any actual and relevant interventions and their effectiveness in improving outcomes (if this exists). There is also little mention what key outcomes of interest are in this study or if this will be clarified as part of the co-design process.

Author response: We thank the reviewer for their comment. In an above response we have further elaborated on how, in co-design, it is not possible to define the intervention. Following this, once the intervention is developed and described in a program logic model, the specific key outcome with the be able to be specified. We have addressed this in two parts below.

1) It would be useful to outline the research around the development of any actual and relevant interventions and their effectiveness in improving outcomes (if this exists).

Author response: We thank the reviewer for the suggestion. We have added some further information from previous Ophelia studies that have evolved into randomised control trials. See Page 9, Lines 204-207.

2) There is also little mention what key outcomes of interest are in this study or if this will be clarified as part of the co-design process.

Author response: We thank the reviewer for the suggestion. As noted above, due to the co-design and that the interventions have not yet been developed, we cannot specify key outcomes. The outcomes will align with the study's aims, i.e., to apply a health literacy development approach (the Ophelia process) to the NSW prison system to co-design actions and solutions to improve health service quality and health outcomes for people in prison. We do have expectations of that process and output aims from the first phase that will be related to engaging with various stakeholders and co-designing health literacy interventions. We anticipate these will include many interventions that may or may not be implementable in/across prisons. The key outcomes for Phase 2/3 will emerge from the co-design process and deep engagement with our stakeholders, executive sponsors and governance groups.

Reviewers comment: There are multiple ethical issues when conducting research in prison, including how they will gain informed consent, issues around power differences in prison, building trust for true co-design etc. This is currently missing and would strengthen the protocol.

Author response: We thank the reviewer for the suggestion. We agree there are multiple ethical issues with conducting prison research, which is evident from the four Human Research Ethics Committees that are presiding over this research project (refer to page 24, Lines 506-517). Further, we have detailed the key ethical considerations related to co-design/research in the new section titled "Applying co-design methods in prisons", see Pages 10, Lines 210-226. Further, we have detailed our governance structures as previously mentioned.

Reviewers comment: It would be useful to set the scene with a global focus with a sense of the prison population in Australia. Authors also offer some useful size of the prison population, and it would be useful to understand this as a rate with any meaningful comparison to other states or countries.

Author response: We thank the reviewer for the comment. We have now added a section to introduce the concept of the prison (Page 5, Lines 94-101). We have also added some further details to set a global focus, and state the size of the NSW prison context, please see Page 5, Lines 104-108.

Reviewer comment: Line 80: useful to add a reference to the first sentence.

Author response: We thank the reviewer for the suggestion. We have now referenced this; accordingly, please see Page 5, Line 104.

Reviewer comment: Line 80: it is not usual to describe people as having 'experienced prison' – it is also an ambiguous statement as not clear if the 42,000 figure refers to the prison population size currently or about people who have been in prison that year (as 'experienced' is past tense). Would be useful to rephrase.

Author response: We agree and have now rephrased this sentence. Please see Page 5, Lines 104-106.

Reviewer comment: Line 87: do people in NSW prisons experience a disproportionate burden compared with other prisons in other states? Or compared with the general population? Worth clarifying this sentence.

Author response: We thank the reviewer for the comment. We clarified this to be a comparison with the general population. Please see Page 5, Line 114.

Reviewer comment: Line 92: how will it inevitably impact the overall health of the general population?

Author response: We thank the reviewer for highlighting this for us. We have removed this sentence from the manuscript as our study focuses on people's health within the prison context. Please see Page 6, Lines 119-121.

Reviewer comment: Line 95: ‘People in NSW prisons are incarcerated in 34 prisons…’ – a bit of a circular sentence (the sentence is like saying - people in prison are in prison in prisons). Worth rerevising to something like ‘There are 34 prisons in NSW state …’

Author response: Thank you to the reviewer for bringing this to our attention. We have revised this statement as suggested. Refer to Page 6, Lines 123-124.

Reviewer comment: Line 103: ‘However, access to these services has been observed to vary from site to site.’ - this sentence needs more information and a reference

Author response: We thank the reviewer for the comment. We have added further information following this sentence and a reference where appropriate. Please see Page 6, Lines 131-135.

Reviewer comment: Lines 120-132: you state there have been two studies examining health literacy in prison – but only describe one. Would be more comprehensive to describe both considering they are the only two.

Author response: Thank you for the suggestion. We have described the other study and noted the limitations detailed by the author. Please see Page 7, Lines 157-162. To note, we have made a slight change to accommodate the description to introduce the second study. Please see Page 7, Lines 163 and 164.

Reviewer comment: Lines 171-180: Two advisory groups have been established ‘due to the ethical considerations and requirements …’. But it is not clear what these ethical considerations and requirements are. How will you ensure these groups are supported and meaningful co-design and participation is reached (relates to my more substantial points too).

Author response: We thank the reviewer for the suggestion. We believe we have addressed this in earlier comments. Further, we have added some greater detail on Page 11, Lines 247-250.

Reviewer comment: Lines 186: Although Table 2 is useful, it would be helpful to the reader to have a sentence or two outlining the HLQ briefly rather than having to engage with a whole table.

Author response: We agree and thank the reviewer for their suggestion. We have updated Table 2 (see Page 13, Line 289) to provide a more straightforward explanation and moved the previous version of Table 2 to the Supplementary files (see Supplementary File 1). Moreover, we have added further description of the HLQ in the text; see Pages 13, Lines 283-288.

Reviewer comment: Lines 187-189: although the HLQ data has been collected and reported elsewhere, it seems important to give a summary of this and rationale for re-analysing in this study.

Author response: We appreciate this feedback. To avoid repetition, we added a '(described above)' to refer readers to the introduction. Moreover, we have provided a rationale for re-analysing the HLQ data in this study, see Page 13, Lines 293-299.

Reviewer comment: Lines 190-197: it is not clear who are the community members? Who will be involved in developing the series of vignettes? How will vignettes be developed? Much of this information is in the primary data collection section as well – so there is also some repetition.

Author response: We thank the reviewer for the comment. We have removed the term community member on Page 11, Lines 241, added some description as intended on Page 11, Lines 252-253 and clarified this to be 'people in prison' on Page 14, Line 299. We have added further information about the development of the vignettes; see Page 14, Lines 300-312. Further, we hope the repetition has been reduced as the whole methods section has been restructured following feedback from another reviewer and we deleted the vignette development information previously described in the 'Cluster analysis' section (see Page 19&20, Line 405-409).

Reviewer comment: Line 201: Who are the other stakeholders?

Author response: Thank you for the comment. When we refer to "other stakeholders' we mean individuals who are not currently involved in the project. As we co-design interventions, new stakeholders (e.g., clinicians or people in prison at a particular site or individuals from an external organisation) may become relevant and key stakeholders in the next phase. Phase 2 of the process details (see Pages 20 & 21, Lines 421-436) the relevance of other stakeholders.

Reviewer comment: Lines 206-211: need to outline a bit more about a logic model and who/how it will be developed; what is the PDSA cycle and why – need a reference.

Author response: We thank the reviewer for the suggestion. We have added more information about the program logic model, its development, and PDSA cycles and referenced these where appropriate on Page 20&21, Lines 429-436.

Reviewer comment: Lines 224-225: 10 interviews lasting just 30mins with prisoners seems very short to provide the in-depth basis for vignettes etc. This needs further justification.

Author response: To clarify, the semi-structured interviews are not the only source of data for the vignettes. The cluster analyses of previously collected HLQ data will provide the basis of vignettes with the semi-structured interviews used to provide real-life experiences to aid the development of vignettes. We have provided further details and clarified this in the manuscript, please see Page 14, Lines 299-305 and Page 16, Lines 334-344.

Reviewer comment: Line 255: why will there be a separate cluster for individuals who identify as Aboriginal, Torres Strait Islander or both?

Author response: Thank you for bringing this to our attention. It is well known that Aboriginal and Torres Strait Islander people in Australia have differing cultural views about health. Moreover, there is a

10-fold over-represented of Aboriginal people in NSW prisons. The second analysis will be undertaken in recognition of potential differences in their cultural views that may result in different ideas for actions and solutions. Further, we sought advice from our Aboriginal Community Reference Group, who supported a secondary analysis to be undertaken. From the data analysis perspective, mixing the 2 groups may lead to drowning out potentially important and unique characteristics of Aboriginal people, given the overwhelming majority of the responses (and variance) is non-Aboriginal. Finally, interventions and actions for Aboriginal people tend to most effective when derived (co-designed) with the full milieu of Aboriginal culture and unique causes of inequity included in focused analyses. We have added a rationale for this in the manuscript; please see Page 19 and Lines 400-404.

Reviewer comment: Potential limitations section – there are several limitations missing, including those relating to my more substantial points (i.e. limits of co-design in prison, extent of interventions which are feasible in prison, lack of focus on outcomes…)

Author response: We thank the reviewer for the comment. We have revised the limitation section. Please see Page 23, Lines 491-496.

Reviewer comment: Abstract - This would require revisions in line with the comments made already.

Author response: We have revised the abstract in line with the major changes to our manuscript.

Reviewer: 2
Comments to the Author:
Thank you for the opportunity to review this protocol. The care and attention taken to ensure that language does not focus on deficits, but the strengths of the aims of this project, are well constructured. The purpose, approach and potential outcomes of this study are clearly focu

Author response: We thank the reviewer for their time and comment in relation to this manuscript.

Reviewer: 3
Comments to the Author:
Dear Editor and authors,
many thanks for inviting me to review this protocol, which I think addresses a timely issue in the context of HL and prisons. Please see my individual comments attached as a file, I wouldnt say this is necessarily a minor or major revision, but a revision in any case. Good luck with finalising the text and kind regards!

Author response: We thank the reviewer for their time and comments. We have addressed their additional comments below.

Title:
Reviewer comment: I find the second part of the title as of “A protocol for..” a bit confusing and it misses a “the”, doesn’t it? I understand you want to include Ophelia in the title, but at the same time its quite long then. Usually would only say “a study protocol” or sth similar – it also doesn’t say what you intend to optimize for Ophelia.

Author response: We thank the reviewer for the comment and agree it is missing a ‘the’. We have updated the title to include this, which now reads “Applying a co-design health literacy development

approach for diverse health services in Australian prisons: A protocol for the Optimising Health Literacy and Access (Ophelia) process". This is noted on Page 1, Line 2 of the manuscript.

Abstract

Reviewer comment: What are HL-informed interventions, is that different from HL-interventions?

Author response: Thank you for highlighting this. We have now reframed this wording to help define our intended meaning. This has been defined in the abstract (Page 2, Lines 29-30) and has been defined in the manuscript at first use on Page 12, Lines 262-263.

Reviewer comment: Can it be assumed in the abstract that any reader knows Ophelia?

Author response: We appreciate you bringing this to our attention. We agree that it cannot be assumed and have removed this initial phrase. Further we have moved the first mention to the aim statement in the abstract (Page 2, Lines 35) and stated it to be Ophelia Phase 1 to assist potential readers (Page 2, Line 40)

Reviewer comment: Whats the difference between needed and wanted?

Author response: We agree and thank you for the comment. We have now removed this phrase and replaced it with 'fit-for-purpose' (Page 2, Line 31).

Introduction

Reviewer comment: Whether, in the prison context, you refer to individual HL, organizational HL or a mix of both?

Author response: Thank you for bringing this to our attention. To clarify for the reviewer, our definition of health literacy is that of individuals and communities (including organisations). These are clarified out intended meaning on page 7, Lines 143-152. Further, we introduce and define health literacy development on page 8, Lines 175-182. This includes the concept of an enabling environment, which, in our opinion, would encompass organisational health literacy.

Reviewer comment: How HL is actually intended to / may be supported in the prison context, e.g. do imprisoned people have access to health info?

Author response: We appreciate the comment. Our 2021 study revealed that people in prison have lower scores across all nine of the HLQ scales. We intend to 'improve' health literacy by applying a health literacy development approach through operationalising the Ophelia process in the NSW prison context. This is novel, and we hope it will reveal a wide range of actions and solutions to improve health literacy. Further, to clarify, people in prison have access to health information and services. We have described the health services provided in the NSW prison context on Page 6, Lines 127-141.

Reviewer comment: Whether prisons have an interest and or obligation to work towards improving HL or related concepts?

Author response: Thank you for this comment. Prisons and health authorities in New South Wales are governed separately; however, both organisations inherently work together (see Page 6, Lines 124-129). Both organisations have identified health literacy as one of the key concepts in the joint Healthy

Prisons framework (available here: https://www.nsw.gov.au/sites/default/files/2023-11/NSW%20Healthy%20Prisons%20Framework%20for%20Action%202023.pdf). To clarify, this shows the interest of both organisations to work towards improving health literacy but does not oblige them to do so. Justice Health NSW's interest and commitment to improving health literacy in NSW prisons is evident in our co-author, Wendy Hoey, Justice Health NSW Chief Executive, partnership, involvement, ongoing sponsorship of the project and co-authorship of this protocol manuscript.

Reviewer comment: Examples for previous health interventions for imprisoned people (in AUS/NSW) and to what extent these worked well or not?

Author response: We appreciate the reviewer's comment. We have added some details on reviews that highlight health interventions in prisons (see page 8, Lines 172-174). Given the paucity of data (quality and quantity) of health interventions in prison including their implementation, we have briefly discussed this (see page 8, Lines 169-172). Moreover, despite some interventions being implemented, health disparities continue to perpetuate in this population. Further, to our knowledge, this is the first health literacy development approach that has been used to design interventions in prisons.

Methods

Reviewer comment: What does "consultation" mean in concrete for the protocol dev?

Author response: We thank the reviewer for the comment. To clarify, the project team undertook consultation by formally seeking advice and discussing planned study activities with differing stakeholders before seeking ethical approval. We believe we have addressed this further throughout our revised patient and public involvement section (see page 11&12, Lines 233-274).

Reviewer comment: Its not clear if the HL needs assessment with n=471 has already been done in 2021?

Author response: Thank you for the comment. To confirm, the health literacy needs assessment with n=471 has previously been undertaken. This was undertaken in 2020-21 by Justice Health NSW across 14 metropolitan prisons in New South Wales (please refer to Page 13, Line 291-295). The findings of this study have been published and are available here: https://bmcpublichealth.biomedcentral.com/articles/10.1186/s12889-023-16464-3).

Reviewer comment: Who are community members?

Author response: We thank the reviewer for the comment. We have removed the term community member on Page 11, Lines 241, added some description as intended on Page 11, Lines 252-253 and clarified this to be 'people in prison' on Page 14, Line 299.

Reviewer comment: I wonder if the study design section could be adjusted, it partly reads like you explain the entire study here, and at the same time it may not give a more general impression of what kind of study this is, the theoretical approach underlying this study, etc. I understand you want to say "this is an Ophelia study" but then why wouldn't you use the last part from the introduction to outline the study design?

Author response: We are grateful for the reviewer's suggestion. We have revised and restructured the manuscript's whole method and analysis section. This has included revising the structure of the paper

and clarifying the study design (please see Page 12 and Lines 275-277). Further, we have reorganised the methods to align with the phases of the Ophelia process; please see Pages 11-21, Lines 232-443.

Reviewer comment: I am unclear about the aspects as of program logic model and implementation and evaluation of HL actions. If I'm not mistaken, you do not provide any details on how you will operationalize these steps because the methods section ends with data analysis.

Author response: Thank you for the suggestion. We have added more information about the program logic model, its development, quality improvement cycles and referenced these where appropriate on Pages 20&21, Lines 429-436.

Reviewer comment: I cant find details on how you approach (recruit) study participants for the various phases, particularly the interviews and workshop and here, imprisoned people – how do you approach them and what kind of participation do you expect?

Author response: We thank the reviewer for the comment. We have added these details at multiple points throughout the revised manuscript. This includes:

- Page 16, Lines 335-344.
- Page 17, Lines 358-363
- Page 18, Lines 372-385

Reviewer comment: Workshops: can you be more concrete on how you "generate ideas"?
Author response: We thank the reviewer for the suggestion. We have refined the sentence that introduces the guiding questions for clarity and included further details on how the workshops/yarning circles are facilitated to generate ideas (Page 18, Lines 372-385).

Reviewer comment: Have you explained somewhere in the protocol what yarning cycles are?

Author response: We thank the reviewer for the comment. We have previously defined yarning circles. This is defined and detailed on pages 17&18, lines 347-350. This reads '*(i.e., a conversational form of Aboriginal data collection where researchers listen "to participants' stories about their lived experiences, feelings, thoughts and ideas on the research topic" (70, p. 38))*'.

Reviewer comment: Limitations: your intended study implies quite some active participation of the various target groups, right? If so, do you not expect any challenges with really achieving this active participation?

Author response: Thank you for the suggestion. We have revised the limitation section. Please see Page 23, Lines 491-496.

Additional changes arising from the revision process.

Upon revising the protocol, we have updated the statistics for the NSW prison population to be the latest available on Page 5, Line 106-108 and referenced these appropriately. We have also refined two sentences on Page 8, Line 168-172. Further, to help potential readers we have used sub-headings for the phases of the Ophelia process and methods to improve this section's navigation. This includes:

- Page 11 and 12, Lines 233-276, Patient and public involvement (as requested). We have now split the previous protocol development and project governance section into two separate headings.
- Page 13, Line 278, sub-heading titled “Phase 1: Identification of local strengths, challenges, and issues”
- Page 19, Line 389, sub-heading titled “Cluster analysis”
- Page 20, Line 410, sub-heading titled “Ideas generation and yarning circles”
- Page 20, Line 421, sub-heading titled “Phase 2: Select, plan and test health literacy actions”
- Page 21, Line 437, sub-heading titled “Phase 3: Implement, evaluate and improve health literacy actions”

Moreover, upon resubmitting the protocol, we have been requested to make some minor changes to the manuscript by the editorial office. This includes:

- Removing the figure embedded in the manuscript. Figure 1 and subsequent legend has now been removed (see Page 9 Lines 194-196). We have indicated where we believe Figure 1 would be best placed within the manuscript by the addition of “*[INSERT FIGURE 1 HERE]*” on Page 9, Line 194.
- The inclusion of figure legends at the end the main document file. See page 38, Lines 844-847.
- Addition of the guarantor name in the Contributorship statement. See page 25, Line 550.

VERSION 2 - REVIEW

**Reviewer**	**3**
**Name**	**Lander, Jonas**
**Affiliation**	**Hannover Medical School, Institute for Epidemiology, Social Medicine and Health Systems Research**
**Date**	**06-Dec-2024**
**COI**	

Dear Editor and authors, according to the revisions made for this manuscript, I do not have further questions at this stage. However, I encourage the authors again to shorten the title, it has 25 words now which is quite a lot, as you know title length is an important aspect when potential readers decide whether or not to read your study.

Kind regards, reviewer
